# Laboratory Markers of Impaired Erythropoiesis in Early Diagnosis of Perioperative Anemias

**DOI:** 10.17691/stm2025.17.2.05

**Published:** 2025-04-30

**Authors:** N.B. Teryaeva, O.K. Kvan, Yu.V. Strunina, A.S. Kulikov

**Affiliations:** Clinical Laboratory Diagnostician; N.N. Burdenko National Medical Research Center for Neurosurgery, Ministry of Health of the Russian Federation, 16, 4^th^ Tverskaya-Yamskaya St., Moscow, 125047, Russia; Head of the Department of Clinical and Industrial Transfusiology; N.N. Burdenko National Medical Research Center for Neurosurgery, Ministry of Health of the Russian Federation, 16, 4^th^ Tverskaya-Yamskaya St., Moscow, 125047, Russia; Leading Engineer; N.N. Burdenko National Medical Research Center for Neurosurgery, Ministry of Health of the Russian Federation, 16, 4^th^ Tverskaya-Yamskaya St., Moscow, 125047, Russia; Head of the Anesthesiology-Resuscitation Department; N.N. Burdenko National Medical Research Center for Neurosurgery, Ministry of Health of the Russian Federation, 16, 4^th^ Tverskaya-Yamskaya St., Moscow, 125047, Russia

**Keywords:** transfusion therapy, erythrocyte-containing components of the donor blood, anemia, reticulocyte hemoglobin, delta- hemoglobin, red blood cell indices, complete (clinical) blood cell count

## Abstract

**The aim of the study** was to assess the diagnostic value of laboratory indicators of hemoglobin content in reticulocytes for the early detection of predisposition for perioperative anemia in patients with total hemoglobin within the reference range.

**Materials and Methods:**

Observational retrospective single-center continuous cross-sectional study has been carried out to determine the need for transfusion of erythrocyte-containing components (ECC) depending on the values of reticulocyte hemoglobin (Ret-He) and delta-hemoglobin (Delta-He) in patients with unchanged (within the reference range) values of the total hemoglobin. The groups of comparison were formed using the diagnostic Hema-Plot algorithm, under which Ret-He and Delta-He values deviate from the reference range towards greater or smaller magnitudes in various types of anemia.

**Results:**

Deviations from the reference intervals of Ret-He and Delta-He values were observed in 26% of patients not formally meeting the WHO criteria for anemia on admission. Indications for ECC transfusion therapy were more likely to occur in patients who had changes in Ret-He and Delta-He corresponding to the signs of anemias of different genesis according to the Hema-Plot algorithm.

**Conclusion:**

The Ret-He and Delta-He values in patients with unchanged hemoglobin allow for making a decision on the need for ECC transfusion therapy in the postoperative period.

The differences between the groups formed on the basis of Ret-He and Delta-He deviations from the reference values are in line with the diagnostic signs of anemias of various origins. They also allow one to discuss the variants of impaired erythropoiesis at the very early stages of the disorder and the risk of anemia development in patients with formally unchanged total hemoglobin levels.

## Introduction

Overcoming anemia in surgical practice remains a vital problem despite a long history of the issue. Anemia untreated before surgery is associated with complications both during the operation and in the postoperative period, resulting in a high risk of intra- and postoperative lethality [1–6].

Transfusion of erythrocyte-containing blood components (ECC) necessary in some cases as a life-saving tactic involves, however, additional risks [1–4, 6]. Diagnosis and treatment of anemia at the preoperative (better prehospital) stage is recognized to be the only safe and effective treatment strategy in the international clinical practice [2–5, 7, 8].

Anemia in surgical patients is not only a source of threatening complications but a sufficiently common event [4, 5, 9, 10]. This can be easily explained: pathogenetic mechanisms of surgical diseases and anemia often intersect in oncological patients, they are aggravated by neoplastic and paraneoplastic processes and a number of therapeutic factors [4, 5, 10–12]. At the same time, the nature of anemia is not always evident in the complex of pathological processes, and iron-deficient states among them are a frequent and not the only cause [4, 6, 10, 13]. Differential diagnosis of anemia has been improved for decades, however, the pathogenetic background of inflammation and/or neoplastic processes imposes certain limitations on the interpretation of laboratory data, reduces their specificity, and makes the procedure of establishing final diagnosis difficult [6, 14–16], although the main disease may often require rapid surgical intervention.

Thus, to treat anemia in patients at the preoperative stage, it is important to design an examination algorithm, which will provide the opportunity not only to diagnose anemia fast and easily but establish or at least propose its cause. From this point of view, a diagnostic Hema-Plot algorithm attracts close attention as it may give a hint to the origin of anemia employing complete blood count (CBC) without additional laboratory tests [15]. According to this algorithm, the differential diagnosis is based on the simultaneous assessment of reticulocyte hemoglobin content (Ret-He) and the delta-hemoglobin (Delta-He) estimates. The latter represents the difference between the reticulocyte and erythrocyte hemoglobin content and characterizes thereby the efficiency of hemoglobinization of the erythroid cells at presentation. The results of our previous study [17] allowed us to suppose that the application of this algorithm is justified not only for assessing the nature of already established anemia [18] but also in cases when total hemoglobin concentration remains within the reference ranges. It has been previously suggested [4] that formal compliance with the criteria of anemia diagnosis (120 g/L for women and 130 g/L for men) does not ensure clinical well-being [4].

**The aim of the study** is to assess the feasibility of using the mentioned algorithm for revealing patients prone to the development of perioperative anemia among those in whom traditional screening fails to suspect the pathology.

## Materials and Methods

Case reports of patients surgically treated at N.N. Burdenko National Medical Research Center for Neurosurgery have been retrospectively reviewed. 8216 neurosurgical patients (3680 men and 4536 women) of various nosologies were included in the study: brain neoplasms (gliomas, meningiomas, neurinomas, metastatic lesions); traumatic brain and spinal injuries; spinal stenosis and brain blood vessel abnormalities. The need for planned surgical intervention and absence of anemia as defined by WHO criteria on admission (values of total hemoglobin over 120 g/L for women and 130 g/L for men) were the criteria for inclusion into the study [4, 18]. Severe concurrent diseases in decompensation stage, chronic infectious processes at the exacerbation stage, oncological diseases not associated with CNS injury, hemoglobinopathy were the exclusion criteria.

**Figure 1.**
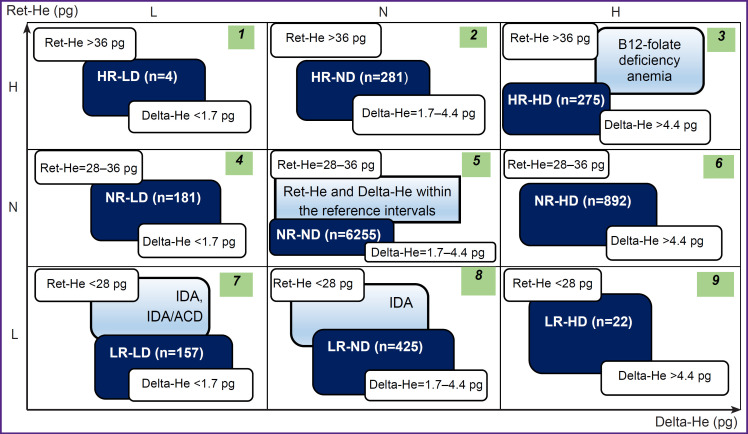
Various combinations of deviations from the reference range for Ret-He and Delta-He values in the Hema- Plot diagram Black on the green background — a serial number of the square in the anemia differential diagnosis diagram, black on the blue background — anemias of various genesis in the study [15], white on the dark blue background — various combinations of deviations in Ret-He and Delta-He values from the reference values; IDA — iron-deficiency anemia, ACD — anemia of chronic diseases, R — reticulocyte hemoglobin (Ret-He), D — delta-hemoglobin (Delta-He), H — values higher than reference intervals, L — lower than reference intervals, N — within the reference intervals

All examined patients were divided into 9 groups based on the results of the complete blood count on admission depending on the values of Ret-He and Delta-He (Figure 1) [15, 17]. According to the Hema-Plot algorithm, axes of abscissas and ordinates correspond to these values. Each axis includes intervals of low, middle (within the reference range), and high values, the diagram area appears to be divided into 9 squares, and, as the result, anemias of various origins are also positioned in different squares, as shown in the study of Weimann et al. [15].

The following designations were adopted for the group names: R — reticulocyte hemoglobin (Ret-He), D — Delta-He, H — values higher than the reference intervals, L — lower than the reference intervals, N — within the reference intervals. Respectively, groups with relatively high Ret-He were denoted as HR, with relatively low Ret-He as LR, NR — within the reference values. Similarly, the designations for Delta-He were as follows: HD — high values, LD — low values, ND — within the reference values, which were equal to 28– 36 pg for Ret-He, and 1.7–4.4 pg for Delta-He.

Then, the number of ECC transfusions performed in the intra- and postoperative period were compared in each of the formed groups.

A complete blood cell count was performed using Sysmex-XN-1000 analyzer (Sysmex, Japan) with optical Ret-He determination and Delta-He calculation. The results of complete blood tests, on admission before any diagnostic and therapeutic procedures, have been analyzed.

**Statistical data processing** was performed using a programming R language (v. 4.2.1) in the integrated development environment RStudio Server (v. 1.3.1093). The Shapiro–Wilk test was used to determine the compliance of the sample with normal distribution. The Mann–Whitney test was used to analyze statistical hypotheses on the difference in the distribution of quantitative variables. The results are presented in the form of a box plot, the null hypothesis in the statistical tests was rejected at the significance level of p<0.05.

## Results

Among 8216 patients included in the study, the values of hemoglobin concentration were not lower than 120 g/L for women and 130 g/L for men, according to the complete blood test. ECC transfusion during the inpatient treatment was required for 232 patients and was not required in 7984 cases. Results of Ret-He and Delta-He within the reference range appeared in 6058 of 8216 patients (74%), and in 2158 cases (26%), their deviations towards the greater or smaller values were observed in one or both indicators.

**Figure 2.**
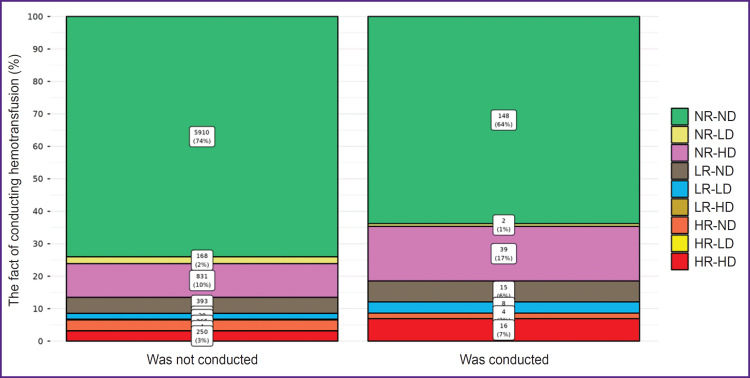
Groups of patients who received and did not receive the transfusion therapy of erythrocyte-containing components R — reticulocyte hemoglobin (Ret-He), D — delta-hemoglobin (Delta-He), Н — values higher than the reference intervals, L — lower than the reference intervals, N — within the reference intervals

Among those who underwent ECC transfusion (Figure 2), patients with Ret-He and Delta-He values within the reference range (NR-ND square of the Hema- Plot; see Figure 1) made up 64%. Among the patients who did not require ECC transfusion, the values within the reference range (NR-ND) were observed in 74%.

Among the patients whose Ret-He or Delta-He values of both indicators differed from the reference, 36% required ECC transfusion and 26% did not need it.

**Figure 3.**
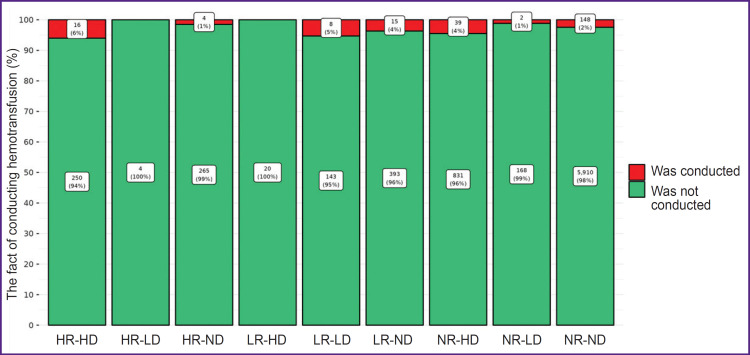
The number of transfusions of erythrocyte-containing components (absolute number and %) conducted in the groups formed on the basis of Ret-He and Delta-He value deviations from the reference intervals in the Hema-Plot diagram R — reticulocyte hemoglobin (Ret-He), D — delta-hemoglobin (Delta-He), Н — values higher than the reference intervals, L — lower than the reference intervals, N — within the reference intervals

The comparison of the groups, formed on the basis of the deviated values of Ret-He and Delta-He from the reference range, by the number of the performed transfusions is presented in Figure 3. Maximum (in percentage) number of ECC transfusions (6%) fell on HR-HD group (square 3 of the Hema-Plot algorithm), which is statistically significant in comparison with the NR-ND group at p=0.002 (see Figure 3 and the Table). In the LR-LD group (square 8), the need in transfusions was 5%, in groups NR-HD and LR-ND (squares 7 and 9) — 4%. In NR-ND group (square 6, all values are within the reference range) the transfusion rate was 2% (statistically significant as compared to the HR-HD and NR-HD groups). The minimum transfusions (1%) were observed in the HR-ND group (square 2). Two groups, in which ECC transfusions were not registered, appeared to be non-relevant according to the number of patients: HR-LD and LR-HD (of 8216, 4 and 20 patients, respectively).

**Table:** The levels of significance obtained by a pairwise comparison of the groups by the number of the conducted transfusions of erythrocyte-containing components

Groups for pairwise comparison		The level of significance in pairwise comparison
HR-HD	HR-ND	0.006
HR-HD	NR-LD	0.013
HR-HD	NR-ND	0.002
HR-ND	LR-LD	0.033
HR-ND	NR-HD	0.026
NR-HD	NR-LD	0.050
NR-HD	NR-ND	0.001

## Discussion

The results of the study performed have shown that deviations from the reference values of Ret-He and Delta-He were in a quarter of cases (26%) among patients with total hemoglobin within the reference range according to the complete blood cell count at admission and formally not meeting the WHO criteria for anemia (see Figure 1). It turned out that during the hospital stay, indications for the ECC transfusion therapy in these patients occurred more frequently (36% in total) than in patients with unchanged (within the reference ranges) Ret-He and Delta-He (26% in total).

The differences between the 9 Hema-Plot-based groups [15] are not quantitatively great and not always statistically significant (see the Table), but logical: the greater number of ECC transfusions was required in the squares that could match various types of anemia, according to the algorithm. Thus, most transfusions (6%) were in the HR-HD group (square 3); according to the algorithm, it corresponds to B12 folate deficiency anemia (B12A). There were 5% of ECC transfusions in the LR- LD group (square 7) that included anemias of chronic inflammatory diseases (ACD) and ACD along with iron- deficiency anemias (IDA), according to the algorithm. In the LR-ND group (square 8, as per algorithm — IDA without ACD), the probability of transfusions in our investigation was 4%. Among the patients with Ret-He and Delta-He within the reference values (no anemia), the ECC transfusion rate amounted to 2%, which is statistically significant in comparison with the HR-ND group.

Thus, the deviations of Ret-He and Delta-He from the reference ranges can be assumed to be the very early signs of impaired erythropoiesis and indicate the upcoming anemia development even if the level of hemoglobin remains unchanged. According to our data, 5.2% of the total number of patients operated on were admitted to the hospital with the predisposition to the IDA development (LR-ND group) and 3.3% with the predisposition to B12A development (NR-HD group). This assumption must be verified using conventional laboratory anemia markers.

According to the logic of the Hema-Plot algorithm, the LR-LD group (square 7) must represent patients prone to the ACD development. At the same time, an inflammatory process is often associated with oncological and some other surgical diseases [19–21]. Therefore, it is in the LR-LD group that inflammation makes an essential contribution to morbid process and is able to significantly affect common iron status. Therefore, it seems appropriate to examine nosological forms and courses of the illnesses in patient groups. The necessity in the ECC transfusion for LR-LD patients was rather great (5%).

Special attention should be paid to Ret-He and Delta- He values (НR-ND, NR-HD, NR-LD, squares 2, 4, 6), as data were not reported in Weimann’s study [15]. The squares were empty. In our study, patients of this kind are rather numerous: HR-ND — 3.4%, NR-LD — 2.2% and NR-HD — 10.9% of total number of patients. The second largest group (after the NR-ND group where Ret- He and Delta-He are within the reference values) was of particular interest. The shift of Delta-He towards high values with the Ret-He within the reference range might be an evidence of rapid destruction of erythrocytes. The assumption is in line with a rather high need in ECC transfusions in this group (4%) as well as that of IDA (LR- ND, square 8) group. The НR-ND and NR-HD groups were characterized by a relatively low need for ECC transfusions: 1%, which is even less than in the NR- ND group. Probably, these groups were characterized by larger hemoglobin content in reticulocytes compared to erythrocytes, which may be interpreted as the compensatory effect, a reaction to hypoxia associated with the main disease. All these proposals definitely need further empirical verification.

Thus, the deviations in the Ret-He and Delta-He values may serve as independent diagnostic signs for surgical patients in clinical decision-making regarding ECC transfusion. If impaired erythropoiesis cannot be diagnosed using conventional criteria, Ret-He and Delta- He values may indicate the probability of perioperative anemia.

## Conclusion

Among neurosurgical patients whose hemoglobin levels according to the complete blood count were within the reference interval and who do not meet the WHO criteria for anemia, deviations in laboratory signs of reticulocyte hemoglobin content (Ret-He) and Delta-He are observed in 26% of cases.

Indications for transfusion therapy of erythrocytecontaining components are most likely to arise in patients whose changes in Ret-He and Delta-He correspond to the diagnostic signs of anemias of various genesis in accordance with the Hema-Plot algorithm.

The results of the study raise the issue of necessity for early detection of susceptibility to perioperative anemia risk in patients for whom traditional screening fails to suspect impaired erythropoiesis. Additionally, they suggest the possibility of anticipating the nature of anemia using Ret-He and Delta-He values without the need for other laboratory tests.
